# Oncological outcomes post transoral robotic surgery (TORS) for HPV-associated oropharyngeal squamous cell carcinoma, a single-centre retrospective Australian study

**DOI:** 10.1007/s11701-024-01910-0

**Published:** 2024-05-28

**Authors:** Belen Kornfeld, Ahmed Taha, Lee Kyang, Hao-wen Sim, Suzannah Dewhurst, Rachael McCloy, Vanessa Chin, Peter Earls, Andrew Parker, Brett Leavers, Dion Forstner, Peter Floros, Julia Crawford, Richard Gallagher

**Affiliations:** 1 Department of Otolaryngology, Head and Neck Surgery, St. Vincent’s Hospital, 390 Victoria St, Darlinghurst, NSW 2010 Australia; 2 School of Medicine, Notre Dame University, 160 Oxford St, Darlinghurst, NSW 2010 Australia; 3 Faculty of Medicine and Health, University of New South Wales, High St, Sydney, NSW 2052 Australia; 4 The Garvan Institute of Medical Research, 384 Victoria St, Darlinghurst, NSW 2010 Australia; 5 Department of Medical Oncology, The Kinghorn Cancer Centre, 370 Victoria St, Darlinghurst, NSW 2010 Australia; 6 Department of Anatomical Pathology, St. Vincent’s Hospital, 390 Victoria St, Darlinghurst, NSW 2010 Australia; 7 GenesisCare, 390 Victoria St, Darlinghurst, NSW 2010 Australia; 8 Department of Medicine, Notre Dame University, Sydney, NSW 2010 Australia

**Keywords:** Transoral robotic surgery, Oropharyngeal cancer, Oncological outcomes, HPV-positive head and neck cancer, Neck dissection, Percutaneous gastrostomy

## Abstract

We present a cohort review of TORS resection for HPV-associated oropharyngeal squamous cell carcinoma (OPSCC) and its associated oncological outcomes spanning a 10-year period. A retrospective case series review was performed of patients undergoing primary surgical treatment for HPV-associated OPSCC through the St. Vincent’s Head and Neck Cancer service from 2011 to 2022. The primary outcomes were to investigate complete resection of the primary tumour, rates of recurrence, and survival analysis. Secondary outcomes included complications, rates of adjuvant therapy, sites of recurrence and rates of percutaneous endoscopic gastrostomy (PEG). 184 patients underwent TORS-based therapy with neck dissection, and guideline-directed adjuvant therapy for HPV-associated OPSCC. Our median follow-up was 46 months. The positive margin rate on final histopathology analysis was 10.9%. Adjuvant therapy was indicated in 85 patients (46%). The local recurrence rate was 10.9% with the majority (80%) of patients recurring in the first 3 years since treatment. The disease-specific survival at 3 years was 98.6% and at 5 years was 94.4%. The 3-year and 5-year OS for the cohort was 96.7% and 92.5%, respectively. The presence of extranodal extension and positive margins were associated with increased risk of recurrence, whereas adjuvant therapy was found to be a protective factor for both overall recurrence and survival. Major complications occurred in 12 patients (6.5%), resulting in one death. This study has demonstrated that primary surgical therapy for HPV-associated OPSCC is a safe and effective treatment modality with low local recurrence and complication rates, and overall survival benefits.

## Introduction

The incidence of oropharyngeal squamous cell carcinoma (OPSCC) has been steadily rising over the past two decades in contrast to other head and neck cancers [1, 2]. Human Papillomavirus (HPV) is now recognised as the main risk factor leading to the development of OPSCC [1–4]. At our institution, 85.3% of cases are HPV-associated [3]. HPV subtypes 16 and 18 are most commonly associated with HPV-associated cancers and in OPSCC, HPV type 16 is the most common HPV subtype, occurring in up to 84% of cases [4–6]. HPV-associated OPSCC has a significantly better prognosis compared to HPV-negative disease irrespective of the treatment modality offered [6–11].

Over the past 20 years, there has been an ongoing argument amongst treating clinicians regarding the best treatment approach for OPSCC. The landscape has now changed with the recognition that HPV-positive disease is sensitive to all modalities of treatment in appropriately selected patients. Weinstein and O’Malley understood the potential of transoral robotic surgery (TORS), and in 2006, they convened a TORS research workshop with a group of likeminded surgeons from across the United States [12]. This collaboration resulted in U.S. Food and Drug Administration (FDA) approval of TORS for clinical use in 2009.

Surgery via a transoral approach in combination with neck dissection for early-stage HPV-associated OPSCC has proven to be as effective in curing disease as primary radiotherapy with or without concurrent chemotherapy. It is now important that all treating clinicians and treatment centres consider the short and long-term side effects and morbidity of each modality of treatment in the management of HPV-associated OPSCC [13–17]. Our aim is to now individualise treatment to avoid long-term morbidity from unnecessary multimodality treatment.

Our campus has significant experience in the application of TORS in the management of tumours of the upper aerodigestive tract, as well as other non-tumour related indications. Between 2011 and 2022, a total of 438 cases have been performed using the da Vinci robotic platform utilising a transoral approach. Since 2011, in appropriately selected patients, TORS has been used as a first-line therapy for the treatment of patients with early-stage HPV-associated OPSCC. Other indications have included non-HPV-associated oropharyngeal tumours (p16 negative SCC, minor salivary gland tumours), supraglottic tumours, hypopharyngeal tumours and parapharyngeal space tumours. In this study, we specifically analysed our 10-year oncological outcomes for patients who underwent primary surgical therapy for HPV-associated OPSCC.

## Methods

### Patients and selection

This study received ethics approval from the St Vincent’s Hospital Human Research Ethics Committee (LNR/17/SVH/282) in accordance with the ethical standards of the Declaration of Helsinki. We performed a retrospective review of the prospectively generated database for patients with HPV-associated OPSCC undergoing primary surgical treatment between 2011 and 2022 at our tertiary medical centre. All patients were presented at the St Vincent’s Head and Neck Cancer Multidisciplinary Team (MDT) meeting prior to surgery. TORS procedures were performed by two robotically trained surgeons. Patients underwent neck dissections concurrently or within 2 weeks of their primary surgery.

Inclusion criteria were early-stage HPV-associated OPSCC (T1-T2) and select T3 tumours deemed surgically resectable at the time of presentation. Both tumour and patient factors were assessed. Patient-specific factors which limited transoral surgical access including limited mouth opening and neck extension, the presence of mandibular tori, and narrow width of the mandibular arch were considered contraindications. Imaging contraindications included abnormal relationship of the carotid system to the tumour, evidence of extranodal spread, or distant metastatic disease. Patients were also excluded if they had neck dissections performed at other institutions or had disease of unknown primary. The specific tumour factors assessed were palatal involvement, depth of invasion and relationship to surrounding structures. Contraindications were extension of the tumour to midline soft palate or extension to within 1 cm of the hard palate as this would result in significant post-operative velopharyngeal insufficiency. Extension beyond the midline tongue base or significant involvement of the lingual surface of the epiglottis was another contraindication as this would impact on long-term swallowing and airway protection. Involvement of the parapharyngeal space or lateral third of the tongue base/glossotonsillar sulcus was a contraindication as to avoid injury to the lingual nerve, hypoglossal nerve or the carotid system.

### Clinical and pathologic features

Tumour staging was determined according to the updated 8th edition of the staging system for malignant head and neck tumours of the American Joint Committee on Cancer (AJCC8) [18]. Those who were initially staged prior to 2018 utilising AJCC7 were restaged according to AJCC8. Data collected included age at time of diagnosis, sex, HPV status (by both p16 staining and HPV ISH testing), smoking status, alcohol consumption, presenting symptoms, clinical and pathologic TNM classification, resection margins, number and locations of lymph nodes involved, and the presence of high-risk pathological features such as perineural invasion (PNI), lymphovascular invasion (LVI), and extranodal extension (ENE).

### Treatment and follow-up

The surgery was performed using the Da Vinci^®^ Surgical Platform with the Si system initially and, from 2014, with the Xi system. The Feyh-Kastenbauer–Weinstein O’Malley (FK–WO) retractor was used to expose the oropharynx. The tumour was resected en bloc with additional resection margins in areas of concern following macroscopic evaluation by the surgeon or from microscopic confirmation via intraoperative frozen section examination. Neck dissection was performed either concurrently or within 2 weeks of the primary tumour resection. Each nodal level was submitted for histopathology separately. Surgical histopathology was discussed at the St Vincent’s Head and Neck Cancer pathology Multidisciplinary Team meeting (MDT) to determine the need for adjuvant therapy. The indication for adjuvant therapy was guided by NCCN guidelines and determined by high-risk features on histopathology including margin involvement, PNI, LVI, number and size of lymph node involvement, and the presence of ENE. Adjuvant treatment included radiotherapy or radiotherapy with concurrent chemotherapy. The radiotherapy dose and fields were individualised for each patient. In cases with involved margins or extranodal extension, concurrent chemotherapy was used. In the case of involved margins, patients received 64–66 Gy in 30–33 fractions to the primary tumour site. Involved nodal levels received 60 Gy and uninvolved levels received 54 Gy in 30 fractions. If the neck was not involved on review of the pathology, radiotherapy was provided to the primary site alone.

Long-term follow-up was scheduled at 3 monthly intervals for the first 2 years, 4th monthly in the third year and 6 monthly thereafter to 5 years. A post-treatment PET-CT with diagnostic CT of the neck and chest was performed at 3 months and then yearly until 5 years.

### Statistical analysis

Descriptive statistics were used to summarise baseline characteristics (age at operation, gender, tumour site and laterality, staging, smoking and alcohol history) and surgical outcomes (margins, ENE, PNI, LVI, complications, major morbidity and mortality, use of adjuvant therapy, PEG dependency). Time-to-event data (OS, disease-specific survival, time to recurrence, time to local recurrence, and time to distant recurrence) were calculated from the date of initial surgery and described using the Kaplan–Meier method. Univariable and multivariable Cox proportional hazards regression were used to compare outcomes by margins, ENE, PNI, LVI, and need for adjuvant therapy, as determined from the initial surgery. Due to the descriptive nature of this study, adjustments were not made for multiple comparisons. Median follow-up time was calculated using the reverse Kaplan–Meier estimator. All statistical analyses were performed using R Statistical Software Version 4.3.1 (R Foundation for Statistical Computing, Vienna, Austria).

## Results

### Patient characteristics

Patient demographics are summarised in Table 1. 184 patients were included in the final analysis with a male predominance (*n* = 152; 82.6%). The average age at time of diagnosis and intervention was 59 years old (interquartile range (IQR) 12). Additional patient demographic characteristics are listed in Table 1.

**Table 1:** Patient characteristics undergoing TORS-based therapy for HPV-related oropharyngeal SCC

Characteristics	No. (%)*
Age (y), median (IQR)	59 (12)
Sex	
Male	152 (82.6)
Female	32 (17.4)
Smoking	
Non-smokers	90 (48.9)
Ex-smokers	69 (37.5)
Current smokers	24 (13)
Unknown	1
Alcohol consumption	
Non-drinkers	66 (35.9)
1–20 grams per day	52 (28.3)
> 20 g per day	61 (33.2)
Unknown	5

*Values are No. (percentage)

### Tumour characteristics and treatment data

Complete tumour clinicopathological characteristics and treatment data are outlined in Table 2.

**Table 2:** Tumour clinicopathological characteristics of patient undergoing TORS-based therapy for HPV-related oropharyngeal SCC

Clinicopathological characteristics	Total n (%)*	Clinicopathological characteristics	Total n (%)
**Sub-site**		**Neck treatment**	
Tonsil Base of tongue (BOT) Glossotonsillar sulcus (GTS)	106 (57.6) 62 (33.7) 16 (8.7)	Ipsilateral MRND Bilateral MRND Ipsilateral SND Bilateral SND Ipsilateral MRND and contralateral SND	49 (26.6) 6 (3.3) 22 (12) 17 (9.2) 90 (48.9)
**Tumour side**			
Right Left	123 (66.8) 61 (33.2)		
**HPV ISH status**			
Positive Negative/equivocal Not tested	160 (87%) 10 (5.4%) 14 (7.6%)		
**Clinical T Stage**		**Clinical N stage**	
T1 T2 T3	103 (56) 67 (36.4) 14 (7.6)	N0 N1 N2 N3	30 (16.3) 138 (75) 15 (8.2) 1 (0.5)
**Pathological T stage**		**Pathological N stage**	
T1 T2 T3	79 (42.9) 97 (52.7) 8 (4.3)	N0 N1 N2 N3	27 (14.7) 139 (75.5) 17 (9.2) 1 (0.5)
**Margins**		**PNI**	
Clear Close (< 3 mm) Positive	45 (24.5) 119 (64.7) 20 (10.9)	Yes No	17 (9.2) 167 (90.8)
**LV**I		**ENE**	
Yes No	53 (28.8) 131 (71.2)	Yes No	39 (21.2) 145 (78.8)
**Adjuvant therapy**		**Type of adjuvant therapy**	
Yes No	85 (46.2) 99 (53.8)	Radiotherapy Chemoradiotherapy	41 (22.2) 44 (23.9)

*Values are No. (percentage)

*ISH* in situ hybridisation, *MRND* modified radical neck dissection, *SND* selective neck dissection; *PNI* perineural invasion, *LVI* lymphovascular invasion, *ENE* extranodal extension

Most of our patients had a tonsillar primary and were T1N1 at time of diagnosis. Only 30 patients (16.3%) had cN0 disease. At the time of resection, surgical margins were evaluated based on macroscopic appearance by the surgeon or intraoperatively by frozen section if the margin was uncertain. Microscopic analysis of final histopathology revealed positive margins in 20 specimens (10.9%). Final histopathology assessment demonstrated a slight overall up-staging of disease where most patients had T2 disease, whereas N stage remained similar. PNI, LVI, and ENE were identified in 17 (9.2%), 53 (28.8%), and 39 (21.2%) patients, respectively. Adjuvant therapy was indicated in 85 (46.2%) patients based on the final pathology. Of these, RT was provided to 41 (22.2%) patients, and CRT was provided to 44 (23.9%) patients.

### Oncologic outcomes

**Fig. 1:**
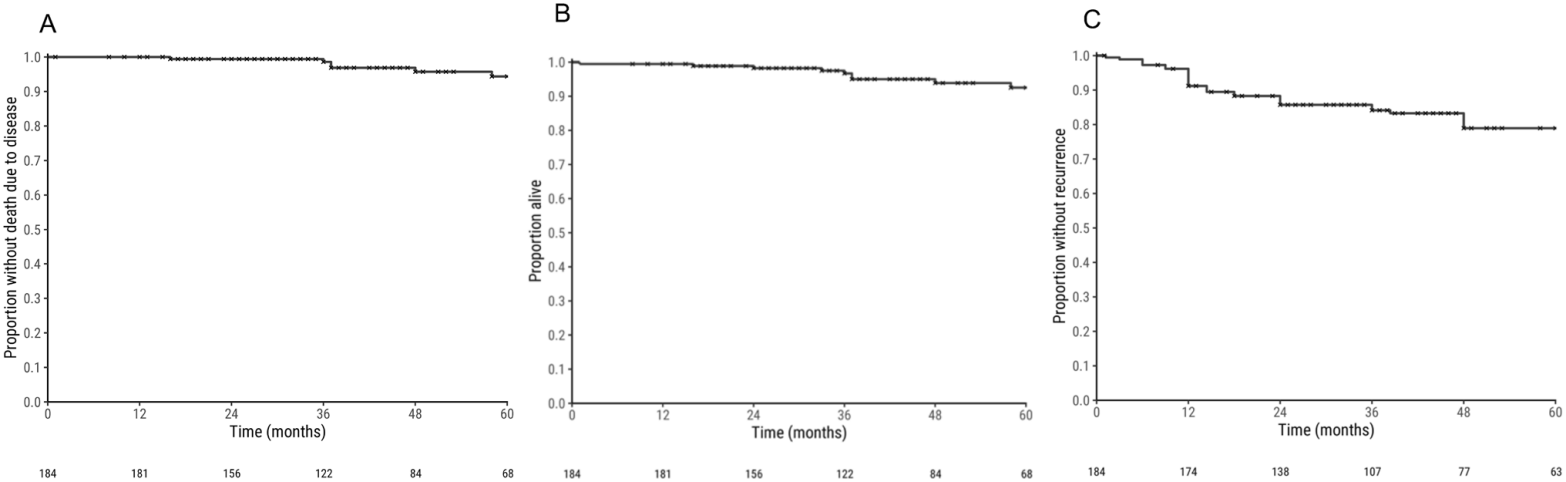
Kaplan–Meier graphs of (**A**) disease-specific survival, (**B**) overall survival and (**C**) time to recurrence for patients with HPV- associated OPSCC undergoing TORS

The median follow-up duration was 46 months [95% CI 42; 52]. Figure 1 demonstrates the disease-specific survival, overall survival and time to recurrence for the cohort. The 3-year and 5-year disease-specific survival were 98.6% (95% CI 96.7–100%) and 94.4% (95% CI 89.9–99.0%), respectively. The 3-year and 5-year OS was slightly lower at 96.7% (95% CI 93.8–99.6%) and 92.5% (95% CI, 87.7–97.6%), respectively. The 5-year overall recurrence-free rate was 78.9% (95% CI 72.4–86.1%). Locoregional recurrence (LRR) occurred in 20 (10.9%) patients, which was mostly seen in tonsillar cancers (7.6%), followed by BOT (2.7%), and then GTS (0.5%). Patients with LRR had slightly lower 3-year and 5-year OS rates of 93.3% (95% CI 81.5–100%) and 73.8% (95% CI 51.2–100%). The majority of locoregional recurrences (80%) occurred within 3 years from treatment. In contrast, 13 (7.1%) had a metastatic recurrence, with 84.6% presenting within 3 years of follow-up.

Three patients died due to locoregional and metastatic disease recurrence and three from disseminated metastatic disease (including pulmonary, hilar, skeletal, brain, and a combination of these sites). Three patients died from unrelated reasons (one from post-operative haemorrhage, one from a cardiopulmonary arrest, and one from a second primary tumour with metastatic disease). There were 4 patients with metastatic disease recurrence that were being treated with palliative intent at time of data collection that were not included as a mortality in this study.

### Prognostic outcomes

Table 3 highlights the results of multivariable analysis of clinicopathological characteristics associated with OS and recurrence for patients with HPV-related OPSCC undergoing TORS. The presence of ENE (HR 2.37, 95% CI = 0.99–5.65, *p* = 0.05) may be associated with an increased risk of overall recurrence but was not a predictor of locoregional recurrence alone (HR 1.64, 95% CI = 0.40–6.72, *p* = 0.49). The presence of a positive margin despite adjuvant therapy was predictive of both locoregional (HR 4.24, 95% CI = 1.26–14.26, *p* = 0.02) and overall recurrence (HR 3.48, 95% CI = 1.47–8.23, *p* = 0.004). In contrast, administration of adjuvant therapy (for ENE, PNI, LVI, positive margins) was a protective factor against both locoregional (HR 0.12 95% CI = 0.03–0.50, *p* = < 0.004), and overall recurrent disease (overall recurrence HR 0.33, 95% CI = 0.13–0.84, *p* = 0.02). Smoking, primary sub-site, and “close margins” (defined as tumour < 3 mm from mucosal edge) were not associated with overall survival (*p* value 0.93, 0.58, 0.21, respectively) in univariable analyses and were not incorporated into the multivariable model.

**Table 3:** Multivariable analysis of clinicopathological features associated with overall survival and recurrence for patients with HPV-associated oropharyngeal SCC undergoing TORS-based therapy

Clinicopathological feature	Overall survival		Locoregional recurrence		Overall recurrence (locoregional + distant)	
	Hazard ratio (95% CI)	P-value	Hazard ratio (95% CI)	P-value	Hazard ratio (95% CI)	P-value
ENE	1.29 (0.23–7.18)	0.77	1.64 (0.40–6.72)	0.49	2.37 (0.99–5.65)	0.05
PNI	3.39 (0.62–18.61)	0.16	1.65 (0.35–7.86)	0.53	2.15 (0.83–5.51)	0.11
LVI	0.24 (0.03–2.08)	0.20	1.42 (0.46–4.34)	0.54	1.76 (0.80–3.84)	0.16
Positive margins	1.81 (0.35–9.41)	0.48	4.24 (1.26–14.26)	0.02	3.48 (1.47–8.23)	0.004
Adjuvant therapy	1.17 (0.25–5.60)	0.84	0.12 (0.03–0.50)	0.004	0.33 (0.13–0.84)	0.02

There were 119/184 (64.7%) patients with “close margins”. Of these, 65/119 (54.6%) did not receive radiotherapy for other reasons (i.e. no PNI, LVI or ENE). There were 4 patients in this cohort (4/65, 6.2%) who were treated with surgery alone and recurred locally. All four patients were initially salvaged. There were two patients who had further distant metastatic disease recurrence. Table 4 describes the recurrences in this cohort and how they were treated.

**Table 4:** Site and treatment of local recurrence in HPV-associated oropharyngeal SCC patients with close margins (< 3 mm) that did not receive adjuvant therapy

Primary site	Site of recurrence	Treatment	Outcome
Tonsil	Ipsilateral GTS	CRT	Successfully salvaged
Tonsil	Ipsilateral tonsil	CRT	Initially salvaged with CRT. Additional distant metastatic recurrence 12 months later to rib salvaged with chemotherapy. Further metastatic disease 2 months later to liver and lung managed with palliative chemotherapy and subsequent mortality.
Tonsil	Ipsilateral soft palate	Sx + RT	Initially salvaged with surgery and RT. Additional distant metastatic recurrence 6 months later to skull base and lung managed with palliative chemotherapy. Palliated at time of data collection
GTS	Ipsilateral tonsil fossa	CRT	Successfully salvaged.

*CRT* chemo-radiotherapy, *Sx* surgery, *RT* radiotherapy

### Complications

Post-operative TORS-related complications occurred in 12 patients (6.5%). One patient (0.5%) had an early post-operative haemorrhage, defined by bleeding occurring less than 24 hours following surgery, resulting in death four days later. Nine patients (4.9%) had a secondary post-operative bleed, defined as bleeding occurring greater than 24 hours following surgery, with all patients being successfully managed either conservatively or with operative management. Salivary fistula occurred in 2 patients (1.1%). PEG insertion for swallowing dysfunction occurred in 10 patients (5.4%) with only 2 patients (1.1%) requiring PEG for greater than 12 months.

## Discussion

This is the largest single-centre Australian cohort treated with primary surgical therapy for HPV-associated OPSCC spanning an extensive follow-up period. The OS of our cohort was 96.7% and 92.5% at 3 and 5 years, respectively. Our disease-specific survival is higher at 98.8% at 3 years and 94.4% at 5 years. Our 5-year LRR was 10.9%, with no significant impact on short-term OS at 3 years (91%) and a less favourable OS at 5 years (71%). Disease-related mortality was rare (*n* = 6) and makes establishing meaningful associations with regard to survival, particularly the impact of locoregional or metastatic recurrence difficult with our available data.

Carey et al. found comparable OS (93.9%) and a lower 5-year LRR (4.5%) in 541 HPV-associated OPSCC patients treated with TORS over 10 years. However, patients with LRR experienced significantly lower overall OS compared with this current study (67.1%) [19]. Nevertheless, this figure is higher than various published studies demonstrating an OS of 50% [20, 21]. Similarly, Brody et al. reported a similar 5-year OS rate as other studies (91.2%), and a recurrence-free survival of 86.1%, though their 5-year OS following locoregional recurrence was significantly higher at 82.1% (95% CI, 63.8–91.7%) [22]. In our study, patients treated with surgery and guideline-indicated adjuvant therapy have demonstrated higher OS than previous publications to date with comparable LRR rates and maintenance of high OS post-recurrence.

An additional benefit of primary surgical management of OPSCC is that it allows for evaluation of high-risk pathologic features that directs treatment decisions such as adjuvant therapy. TORS is associated with increased margin clearance and improved OS in patients with OPSCC than previously described surgical approaches [13, 23]. The definition of a “clear” or “close margin” lacks clarity with guidelines and clinical trials proposing different classifications. Contradictory data exist regarding the significance of margin status on the recurrence rate and OS. In our centre, we recognise that the purpose of TORS is to provide minimally invasive surgery. In many instances, it is not possible to attain a generous margin due to its proximity to important anatomical structures and to preserve swallowing function. Moreover, pathological specimens shrink 15–75% ex vivo, making accurate interpretation difficult [24]. Margin status in our study was recorded as either positive or negative, the closest mucosal and deep margin was also documented. Intraoperative frozen section that returned benign cells was recorded as negative for margin status. In our institution, a positive margin is an indication for adjuvant therapy, whereas a “close margin” is not. Our results compared favourably with other studies demonstrating a low positive margin rate of 10.9%. There was a significant range in the variability of margin status in the literature ranging from 4 to 20% [13, 14, 25]. In our study, patients with positive margins were more likely to have both locoregional and metastatic disease recurrence. However, this did not clearly correspond to a change in OS (*p* = 0.48). This may be a result from our close follow-up and aggressive salvage therapies. This is in contrast to previous studies by Moore et al. and de Almeida et al. that reported no association between final margin status and LRR [13, 26]. Future studies and current clinical trial results may be valuable in determining the optimal margin range to dictate the application of adjuvant therapy.

The significance of additional pathologic prognostic markers on LRR and OS remains controversial. Most studies have not been able to determine an independent association of pathological factors on LRR [27–29]. A recent meta-analysis by Benchetrit et al. highlighted that ENE was associated with an increased risk of distant metastasis and all-cause mortality [30]. Our study supports these results as ENE was an independent prognostic pathologic feature associated with distant disease recurrence (HR 2.37, 95% CI = 0.99–65.65, *p* = 0.05). The presence of poor prognostic pathological markers (ENE, LVI, PNI, positive margin) remains an indication for adjuvant therapy as per NCCN guidelines [31]. Although, adjuvant therapy must be used judiciously as it can cause harmful side effects and may limit chemo-radiotherapy options in salvage therapy [32–34]. For HPV-associated OPSCC, there remains conflicting results in the literature regarding the benefit of adjuvant therapy on the rates of locoregional recurrence and OS rates [35–37]. Although our results suggested limited impact on OS, multivariate analysis showed that treatment with adjuvant therapy decreased rates of both locoregional (HR 0.12, 95% CI = 0.03–0.50 and distant recurrence (HR 0.33, 95% CI = 0.13–0.84, *p* = 0.02), compared to those not requiring guideline-indicated adjuvant therapy. These findings are reinforced by similar cohort studies [19, 26]. Skellington et al. suggested that the addition of adjuvant chemotherapy in surgically treated HPV-associated OPSCC did not improve disease-free survival and was associated with worse OS [38]. Our results emphasise the need to further investigate optimal adjuvant therapy strategies which current de-escalation clinical trials aim to clarify [39–43].

Surgical complications related to TORS remained low, particularly when compared to previous open surgical techniques. Chia et al. summarised the common complications surgeons experienced with TORS in 2013 [44]. In that study, 7.3% (14/190) experienced complications which is lower than rates previously described in the literature 10–30% [45]. Significant life-threatening haemorrhage is of main concern for TORS ranging from 0 to 9%, with bleeding-related mortality occurring < 1% [44, 45]. In our study, our bleeding rate is comparable to previously established data and remained low at 5.4% (10/184). Similarly, perioperative mortality was a rare occurrence (1/184, < 1%). Current PEG dependency rates following TORS is 0–9.5% at 1 year, and 5% long-term [17, 26, 45]. Our PEG dependency rate post-TORS was similar at 5.4% (10/184), with only 1.1% (2/1184) requiring PEG for more than 12 months. Our low complication rate highlights the safe utility of this treatment modality. Given advancement and improvement in technique, these complication rates are likely to further improve in years to come.

We recognise the limitations of this study. First, this is a retrospective analysis which inherently will introduce variability in the analysis and documentation of pathologic variables. However, given our study was conducted at a single institution with two surgeons utilising similar techniques with regards to en bloc TORS resection, neck dissection, and histopathological analysis, there is an element of reciprocity. Due to the limited number of patients that died (9/184), the available data may not be sufficient to establish meaningful associations for survival analysis. Data regarding functional outcomes outside the need for PEG insertion, such as swallowing, and quality of life factors were outside of the primary aims and scope of this study. However, we do recognise that these pose an integral role in the decision-making process in OPSCC treatment. This study adds to the growing body of work that supports TORS as an important oncological treatment option in the management of patients with HPV-associated OPSCC.

## Conclusion

Primary surgical therapy for HPV-associated OPSCC with TORS is a safe and effective treatment modality. Our study demonstrated high disease-free and OS benefits associated with surgical management followed by guideline-indicated adjuvant therapy. This minimally invasive approach has an expanding role in the treatment of these cancers and has revolutionised the surgical approach to OPSCC in the last decade.

## Data Availability

No datasets were generated or analysed during the current study.
